# Single Sequential Trajectory Optimization with Centroidal Dynamics and Whole-Body Kinematics for Vertical Jump of Humanoid Robot

**DOI:** 10.3390/biomimetics9050274

**Published:** 2024-05-02

**Authors:** Yaliang Liu, Xuechao Chen, Zhangguo Yu, Haoxiang Qi, Chuanku Yi

**Affiliations:** 1 School of Mechatronical Engineering, Beijing Institute of Technology, Beijing 100081, China; liuyaliang@bit.edu.cn (Y.L.); yuzg@bit.edu.cn (Z.Y.); 3120215098@bit.edu.cn (H.Q.); yichuanku@bit.edu.cn (C.Y.); 2 Key Laboratory of Biomimetic Robots and Systems, Ministry of Education, Beijing 100081, China

**Keywords:** humanoid robot, vertical jump, trajectory optimization

## Abstract

High vertical jumping motion, which enables a humanoid robot to leap over obstacles, is a direct reflection of its extreme motion capabilities. This article proposes a single sequential kino-dynamic trajectory optimization method to solve the whole-body motion trajectory for high vertical jumping motion. The trajectory optimization process is decomposed into two sequential optimization parts: optimization computation of centroidal dynamics and coherent whole-body kinematics. Both optimization problems converge on the common variables (the center of mass, momentum, and foot position) using cost functions while allowing for some tolerance in the consistency of the foot position. Additionally, complementarity conditions and a pre-defined contact sequence are implemented to constrain the contact force and foot position during the launching and flight phases. The whole-body trajectory, including the launching and flight phases, can be efficiently solved by a single sequential optimization, which is an efficient solution for the vertical jumping motion. Finally, the whole-body trajectory generated by the proposed optimized method is demonstrated on a real humanoid robot platform, and a vertical jumping motion of 0.5 m in height (foot lifting distance) is achieved.

## 1. Introduction

Humanoid robotics is a burgeoning research field that has great potential for technological advancement and human prosperity. Considerable research has been conducted on the walking and manipulation of humanoid robots, which enables a robot to move through complex terrains and perform dangerous and repetitive tasks [1,2,3,4]. To further explore the full potential of humanoid robots and equip them with the ability to execute highly dynamic tasks, researchers are delving deeper into the study of highly dynamic motions, such as vertical jumping.

A high vertical jump is a challenging task, and many researchers have been working on jumping motions using simple mechanisms or simulations [5,6,7,8,9,10,11,12]. In the last few years, few have realized vertical jumping of a real full-scale humanoid robot platform [13,14,15,16,17,18]. Apart from excellent actuation hardware optimization [14,15,19], dynamic motion control is the key to the success of vertical jumping. Among the current jumping motion control frameworks [13,15,16,17,18], a whole-body trajectory can generate an effective jumping motion during the launching phase [6,7,10,12,16,17], and online control algorithms are designed to compensate as much as possible for some motion errors caused by an inaccurate trajectory. Thus, a whole-body trajectory that satisfies whole-body dynamics constraints can reduce the design complexity of the control algorithms for vertical jump. Because of the complexity of the motions and the high degrees of freedom of a humanoid robot, trajectory optimization has emerged as the most widely used method for generating specified motion trajectories.

Trajectory optimization methods are not limited to the field of humanoid robot jumping and can be divided into three categories. (1) Direct use of the complete whole-body dynamics model for planning: This enables the robot to produce satisfactory trajectories for more complex motion tasks [20]. However, this method sometimes becomes intractable, especially when dealing with high-dimensional, complex robot models. (2) Reduced-order models, such as the spring-loaded inverted pendulum (SLIP), flywheel SLIP, and single rigid body model (SRBM), are used to plan jumping motions. Despite the success of SLIP, its point mass model completely ignores angular momentum [16,21]. Jump motions often involve body rotation, for which angular momentum is indispensable. To alleviate this issue, flywheel SLIP is a potential solution [12]. Furthermore, the assumption of massless legs enables the SRBM to successfully render gait and motion generation problems computationally tractable for quadruped robots [22,23,24]. Unfortunately, humanoid robots have more actuated joints in each leg than a quadruped robot, and hence, the assumption of massless legs is easily violated. (3) Another simplified model, known as kino-dynamic [25], uses centroidal dynamics to reduce the complexity of the whole-body dynamics while guaranteeing feasible kinematic motions. Centroidal dynamics efficiently introduce the angular momentum, which is beneficial for the generation of arbitrary jumping motions. Recently, an alternating kino-dynamic optimization planner has been proposed that iteratively solves two optimization subproblems (the centroidal dynamics and kinematics optimization) until they reach consensus, allowing it to effectively solve whole-body motions [10,26,27].

Specifically, alternating kino-dynamic optimization makes it easier to find a locally optimal solution. During one iteration process, one subproblem needs to perform tracking tasks for common variables (the center of mass (CoM); momentum, including both the angular and linear momentum; and foot position) that were optimized by the other subproblem. Each tracking task is treated equally and objectively. Therefore, it has to iterate many times to ensure the consistency of common variables, which is obviously time-consuming. For example, Budhiraja et al. [27] obtained the whole-body trajectory with high consistency using centroidal dynamics and kinematics after 10 iterations. Based on their work, we propose a single sequential kino-dynamic trajectory optimization method that is designed on the basis of an alternating kino-dynamic planner. This method adds cost functions to ensure that both subproblems come to an agreement on the common variables (CoM and momentum), but consistency in the foot position is not required. In this case, the whole-body trajectory is effectively generated by a single sequential optimization, which first solves the centroidal dynamics optimization and then solve the whole-body kinematics optimization, and it is undoubtedly faster than the alternating kino-dynamic optimization planner. Obviously, the optimization solution time will be further reduced so that the whole-body trajectory of jumping motions can be optimized online, and the jumping flexibility of the robot can be further improved.

In addition, the above studies [16,28] focused only on the trajectory optimization of the launching or landing phases, paying little attention to the flight phase. However, to achieve a high jump, the active retraction motion of the leg is essential in the flight phase, which is a problem that remains be solved. More importantly, when searching for the optimal motions for the launching phase and flight phase, it is necessary to know whether the foot is touching the environment and whether any forces are present. In other words, deciding which of the feet should be in contact at a given time is a matter of finding the contact schedule. A traditional approach is to use a soft-contact model, such as a spring–damper contact model [29], to approximate inherently hard contact surfaces. These virtual spring–damper models need to be exceptionally stiff to emulate a real surface. However, the abrupt contact force changes resulting from a foot hitting a rigid surface can impede the optimizer’s performance. Mixed-integer programming has proved to be an efficient tool to efficiently solve contact schedules, gait transitions, and motion [30,31]. It involves assigning contact to surfaces using mixed integers and can be combined with convex or mixed-integer convex dynamic models, and hence, the computation is large and complicated. In this article, the contact sequence can be defined in advance because a jumping motion with contact is relatively simple. Then, the contact sequence is combined with the linear complementarity condition [24,25,32], which can enforce that either the foot must be zero distance from the contact surface or the force must be zero.

**Figure 1:**
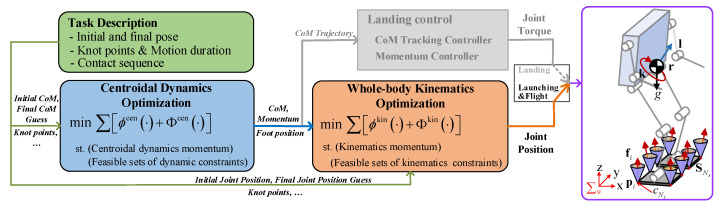
Single sequential kino-dynamic trajectory optimization framework: $\phi^{\mathrm{cen}}\left(\cdot \right)$, $\Phi^{\mathrm{cen}}\left(\cdot \right)$, $\phi^{\mathrm{kin}}\left(\cdot \right)$ and $\Phi^{\mathrm{kin}}\left(\cdot \right)$ represent different cost functions. The contact surface of the foot is $S_{N_{s}}$, $c_{N_{i}}$ denotes the vertice of the contact surface $S_{N_{s}}$, $p_{i}\in R^{3}$ denotes the position of contact point $c_{N_{i}}$, and $f_{i}\in R^{3}$ is a contact force acting on contact point $c_{N_{i}}$. Here, $f_{i}$ is restricted to the inside of the friction cone (shown in purple) or a more restrictive friction pyramid (shown in yellow), r represents the CoM position, the centroidal angular momentum of the robot is k, the centroidal linear momentum of the robot is l, $g\in R^{3}$ is the gravity vector, and $\sum_{w}$ denotes the world coordinate.

In order to solve the above problems and obtain the whole-body trajectory of a vertical jumping motion, including the launching and flight phases, a single sequential kino-dynamic trajectory optimization method is proposed in this work. The brief structure of the optimization method is illustrated in Figure 1. As shown, the task description in the optimization framework includes the initial robot pose (CoM, joint position, and foot position) and final robot pose estimation, motion duration, knot point and re-specified contact sequence, which are important initialization variables for optimization problems. After inputting the initial robot pose, knot point, and other variables into the centroidal dynamics optimization, a set of CoM and momentum values are obtained that satisfy the centroidal dynamics and other feasible sets of dynamic constraints while minimizing the cost function $\Phi^{\mathrm{cen}}\left(\cdot \right)$. The optimized CoM, momentum, and foot position are used as reference inputs for the whole-body kinematics optimization, and the initialization variables such as initial joint angle, the knot points, and other variables in the task description are also required. Then, the whole-body kinematics optimization is solved under the above inputs and kinematic constraints. More importantly, the cost function $\Phi^{\mathrm{kin}}\left(\cdot \right)$ is deliberately added to ensure that both optimization problems come to an agreement on the common variables (CoM and momentum) without requiring consistency in the foot position. Moreover, cost functions $\phi^{\mathrm{cen}}\left(\cdot \right)$ and $\phi^{\mathrm{kin}}\left(\cdot \right)$ are used to penalize the state variables to discourage violent motions. Finally, a whole-body motion trajectory is obtained that satisfies both the centroidal dynamic and whole-body kinematic constraints, enabling the humanoid robot to achieve a high vertical jump. As for the landing phase, the landing controller designed for our team’s previous research [17] is adopted to ensure landing stability. The CoM trajectory that is solved by centroidal dynamics optimization is used as the reference input for the landing controller.

The contribution of this work is summarized as follows:

(1) A single sequential kino-dynamic trajectory optimization framework is proposed to solve the optimal jumping motion problem. The whole-body trajectory is effectively generated by a single sequential optimization, which is an efficient solution.
(2) This optimization framework can generate vertical jumping motions with launching and flight phases, which are essential for highly dynamic motions.

This paper is organized as follows. The single sequential kino-dynamic trajectory optimization method is introduced in Section 2. Section 3 discusses the simulation and experimental results. Finally, the conclusion is presented in Section 4.

## 2. Single Sequential Kino-Dynamic Trajectory Optimization

In this section, the single sequential kino-dynamic trajectory optimization method is introduced. The centroidal dynamics optimization and whole-body kinematics optimization are described separately.

### 2.1. Centroidal Dynamics Optimization

Suppose the contact surface between a humanoid robot (i.e., the robot model shown in Figure 1) and its environment is described by $S_{N_{s}}$, which is a convex polygon with vertices ($c_{N_{1}}$, *…*, $c_{N_{i}}$, *…*, $c_{N_{c}}$) [33,34]. Moreover, the robot’s state can be represented by centroidal linear and angular momentums, and the rates of the linear and angular momentums are determined by the total external force and gravity. Therefore, the equations of motion of the whole robot are given by:

(1) $$\dot{h}=\left[\begin{array}{c} \dot{l}\\ \dot{k} \end{array}\right]=\left[\begin{array}{c} mg\\ 0 \end{array}\right]+\sum_{i=1}^{N_{c}}\left[\begin{array}{c} f_{i}\\ \left(p_{i}-r\right)\times f_{i} \end{array}\right]$$

where *m* represents the mass of the robot.

Generally, researchers use the centroidal dynamics model (Equation (1)) to optimize the CoM and momentum and then combine it with online control algorithms to realize the desired motion [28,35]. To satisfy the real-time demands of an online algorithm, the above optimization approach prioritizes efficiency, often at the cost of trajectory accuracy. In our proposed framework, the centroidal trajectory is used as a reference input for the whole-body kinematic optimization. However, any error in the centroidal trajectory tends to become amplified when mapping the whole-body kinematics. This undoubtedly has a negative impact on highly dynamic jumping motions. Throughout this article, the higher-order dynamic expression of Equation (1) is referred to as the centroidal dynamics model to enhance trajectory accuracy.

The optimization variables for centroidal dynamics optimization include the position r, velocity $\dot{r}$, and acceleration $\ddot{r}$ of the robot’s CoM; the angular momentum k and its time derivative $\dot{k}$; the linear momentum l and its time derivative $\dot{l}$; and the contact force $f_{i}$ and its time derivative ${\dot{f}}_{i}$. Here, we sample all these time-varying quantities at the knot point *N*, with time duration *T* between successive knot points. Additionally, the contact sequence $c_{i}$ is pre-specified, which dictates the sequence in which contact points make and break contact with the ground (touch → off).

The centroidal dynamics optimization can be posed in the following manner:

(2a) $$\min_{h,p_{i}}\sum_{k=1}^{N}\left[\phi_{k}^{\mathrm{cen}}\left(\cdot \right)+\Phi_{k}^{\mathrm{cen}}\left(\cdot \right)\right]$$

(2b) r¨kl¨kk¨k=l˙kl˙kmm∑i=1Ncfik∑i=1Ncp˙ik−r˙k×fik+pik−rk×f˙ik

(2c) $$\dot{r}\left[k+1\right]-\dot{r}\left[k\right]=\ddot{r}\left[k\right]\Delta t,r\left[k+1\right]-r\left[k\right]=\dot{r}\left[k\right]\Delta t$$

(2d) $$\dot{l}\left[k+1\right]-\dot{l}\left[k\right]=\ddot{l}\left[k\right]\Delta t,l\left[k+1\right]-l\left[k\right]=\dot{l}\left[k\right]\Delta t$$

(2e) $$\dot{k}\left[k+1\right]-\dot{k}\left[k\right]=\ddot{k}\left[k\right]\Delta t,k\left[k+1\right]-k\left[k\right]=\dot{k}\left[k\right]\Delta t$$

(2f) $${\dot{f}}_{i}\left[k+1\right]-{\dot{f}}_{i}\left[k\right]={\ddot{f}}_{i}\left[k\right]\Delta t,f_{i}\left[k+1\right]-f_{i}\left[k\right]={\dot{f}}_{i}\left[k\right]\Delta t$$

(2g) $$\sqrt{{\left(f_{i}^{x}\left[k\right]\right)}^{2}+{\left(f_{i}^{y}\left[k\right]\right)}^{2}}\leq \mu {\left(f_{i}^{z}\left[k\right]\right)}^{2},f_{i}^{z}\left[k\right]\geq 0$$

(2h) $$f_{i}^{z}\leq f_{max}c_{i}\left[k\right]$$

(2i) $$\left(p_{i}^{xy}\left[k+1\right]-p_{i}^{xy}\left[k\right]\right)c_{i}\left[k\right]=0$$

(2j) $$p_{i}^{z}\left[k\right]-h_{terrain}\geq 0$$

(2k) $$\ell^{\min}\leq {∥p\left[k\right]-r\left[k\right]∥}^{2}\leq \ell^{\max}$$

(2l) $$F\left(p_{i}\left[k\right]\right)\in S_{N_{s}}$$

The quadratic costs (see Equation (2a)) include a running cost $\phi_{k}^{\mathrm{cen}}\left(\cdot \right)$ and consensus cost $\Phi_{k}^{\mathrm{cen}}\left(\cdot \right)$. Running cost $\phi_{k}^{\mathrm{cen}}\left(\cdot \right)$ is composed of user-defined tack costs, which penalize control variable ${\dddot{f}}_{i}$ to discourage violent motions. It can be defined as follows:

(3) $$\phi_{k}^{\mathrm{cen}}\left(\cdot \right)=\sum_{i=1}^{N_{c}}{∥{\dddot{f}}_{i}\left[k\right]∥}_{Q_{cf}}^{2}$$

where ${∥x∥}_{Q}^{2}$ is an abbreviation of quadratic cost $x^{T}Qx$, Q≥0, and $Q_{cf}\in R^{6\times 6}$ is a force weight matrix. Additionally, square bracket [k] indicates the sampled value at knot point *k*.

Additionally, consensus cost $\Phi_{k}^{\mathrm{kin}}\left(\cdot \right)$ can penalize deviations in the CoM and momentum from the given references. It is expressed as follows:

(4) $$\Phi_{k}^{\mathrm{cen}}\left(\cdot \right)={∥r\left[k\right]{-}^{\mathrm{ref}}r\left[k\right]∥}_{Q_{cr}}^{2}+{∥h\left[k\right]{-}^{\mathrm{ref}}h\left[k\right]∥}_{Q_{ch}}^{2}+\sum_{i=1}^{Nc}\left({∥r\left[k\right]-p_{i}\left[k\right]∥}_{Q_{ce}}^{2}\right)$$

where rref and href are the references obtained by linear interpolation from the initial value and final value estimate, respectively. $Q_{cr}\in R^{3\times 3}$, $Q_{ch}\in R^{3\times 3}$, and $Q_{ce}\in R^{3\times 3}$ are weight matrices.

Centroidal dynamics optimization includes the following constraints:

**Integration constraint**: The constraints of the centroidal dynamics include dynamic (Equation (2b)) and integration constraints (Equation (2c,f)). Note that the forward-Euler integration [36] is adopted to approximate the time derivatives for the variables r, $\dot{r}$, l, $\dot{l}$ and k, $\dot{k}$, $f_{i}$, ${\dot{f}}_{i}$, and Δt indicates time duration.

**Frictional constraint**: The contact force is limited to the inside of the friction cone (friction coefficient μ) to prevent the contact point from sliding, as expressed in Equation (2g). Additionally, the normal forces of the contact force have maximum limits during the launching phase and are always equal to zero during the flight phase. After combining the contact sequence, the constraint on the normal force is expressed by Equation (2h).

**Terrain constraint**: Referring to the complementarity constraint [24,25], we also impose a constraint that specifies that the foot in contact with the ground does not slide during the launching phase. The tangential displacement between two knot points must be zero when the foot is in contact. This constraint disappears during the flight phase, as shown in Equation (2i,j); $h_{terrain}$ represents the height of the ground.

**Distance constraint**: The distance constraint (see Equation (2k)) between the CoM and foot position should be within the predefined range $\ell^{\min}$ and $\ell^{\max}$. The maximum value $\ell^{\max}$ is used to limit the overall length of the robot when stretched, which prevents some joints from approaching singularity. Similarly, the minimum value is used to prevent the robot from contracting too much during the flight phase.

**Vertices constraint**: The relative positions between the vertices ($c_{1}$, *…*, $c_{N_{c}}$) of the foot contact surface $S_{N_{s}}$ are fixed to form a virtual, fixed-shaped sole (Equation (2l)).

This optimization solves the momentum and contact forces in the launching and flight phases. However, it completely ignores the whole-body kinematics of the robot. How to map these to the whole-body trajectory after accurate centroidal trajectories have been obtained is worth researching.

### 2.2. Whole-Body Kinematics Optimization

In this section, the whole-body kinematics optimization is considered to be aligned with the centroidal dynamics optimization results, and the whole-body trajectory for the jumping motion is optimized.

**Figure 2:**
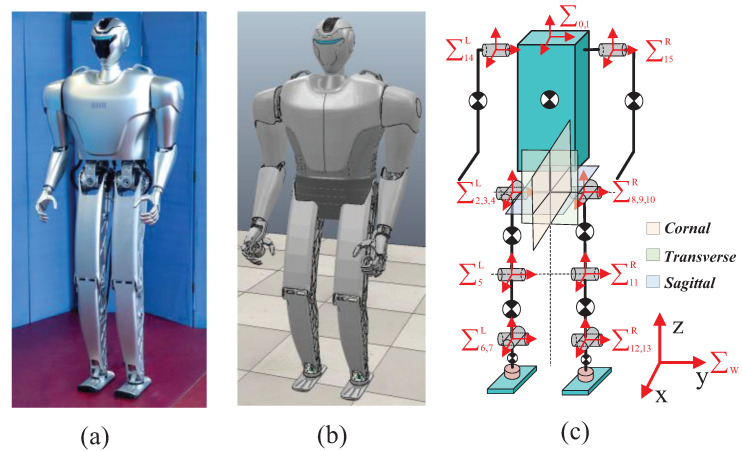
Robot platform used in this article. (**a**) Actual platform. (**b**) CoppeliaSim model. (**c**) Simplified link model.

Because both optimization problems want to be consistent in terms of common variables (CoM, momentum, and foot position), it is necessary to establish a kinematic model. This article mainly describes the calculation process of momentum, but the expressions of CoM and foot position are not described in detail. A humanoid robot can be modeled as a system consisting of n+1 interconnected links and *n* joints, as shown in Figure 2c. The symbol $\sum_{w}$ represents the world coordinate frame, and $\sum_{1}$ represents the float base coordinate frame. The left joints of the robot are represented as $\sum_{2∼7}^{L}$ and $\sum_{14}^{L}$, and the right joints are represented as $\sum_{8∼13}^{R}$ and $\sum_{15}^{R}$. Through the recursive relationship between link *j* and its predecessor link p(j), the spatial speed of link *j* is expressed as follows:

(5) vj=ωjυj=Xpjjvpj+Φjq˙j

where $\omega_{j}$ and $\upsilon_{j}$ represent the angular and linear velocities of link *j*, respectively, in the link coordinate frame; Xpj23j is a 6×6 spatial transform that converts spatial motion vectors from the predecessor $p\left(j\right)$ to *j* coordinates. The matrix $\Phi_{j}$ depends on the type of joint and has full column rank, ${\dot{q}}_{j}$ signifies the velocity vector of link *j* relative to the velocity of its predecessor p(j).

The spatial transform Xpj  j can be composed from the position vector ${\;}^{{\;}_{p\left(j\right)}}p_{j}$, which originates from coordinate frame $p\left(j\right)$ to the *j* coordinate, and the 3×3 rotation matrix ${\;}^{j}R_{p\left(j\right)}$ from coordinate frame $p\left(j\right)$ to *j*:

(6) $${\;}^{i}X_{p\left(j\right)}=\left[\begin{array}{cc} {\;}^{i}R_{p\left(j\right)} & 0\\ {\;}^{i}R_{p\left(j\right)}S{\left({\;}^{{\;}_{p\left(j\right)}}p_{j}\right)}^{T} & {\;}^{i}R_{p\left(j\right)} \end{array}\right]$$

where ${\;}^{{\;}_{p\left(j\right)}}p_{j}$ represents the position vector from coordinate frame $p\left(j\right)$ to the *j* coordinate frame, and $S\left({\;}^{{\;}_{p\left(j\right)}}p_{j}\right)$ represents the transformation of the position vector ${\;}^{{\;}_{p\left(j\right)}}p_{j}$ into a skew-symmetric matrix.

The spatial momentum of the each link can be calculated from the spatial velocity as follow:

(7) $$h_{j}=\left[\begin{array}{c} k_{j}\\ l_{j} \end{array}\right]=I_{j}v_{j}$$

where $k_{j}$ is the angular momentum, $l_{j}$ represents the linear momentum, and $I_{j}$ is the spatial inertia, all in reference to coordinate frame *j*.

Gathering all of the link velocities and joint velocities together, the system Jacobian $J_{j}$ can be defined to give the relationship between the two:

(8) $$v_{j}=J_{j}\dot{q}$$

The total momentum of a humanoid can be calculated by adding up all the angular and linear momentums contributed by the individual link segments, which can be described as follow:

(9) $$h=\sum_{j=1}^{N}{\;}^{i}X_{G}^{T}h_{j}=\sum_{j=1}^{N}{\;}^{j}X_{G}^{T}I_{j}J_{j}\dot{q}=A_{G}\dot{q}$$

where ${\;}^{j}X_{G}$ denotes a transformation matrix that links the *j* coordinate from the centroidal frame, $A_{G}$ is the centroidal angular momentum matrix, and $\dot{q}$ denotes the joint velocity vector.

The total momentum of a humanoid robot is described as follows:

(10) $$h=A_{G}\dot{q}$$

Consequently, the kinematics centroidal momentum is obtained. Next, the whole-body kinematic optimization problem is described in detail. The robot’s states are used to determine decision variables, including joint position q, joint velocity $\dot{q}$, and joint acceleration $\ddot{q}$, which are employed as the control values. Here, we utilize the same knot points *N*, time durations *T*, and contact sequence $c_{i}$. The whole-body kinematic optimization is formulated as follows:

(11a) $$\min_{\;q,\dot{q},\ddot{q}}\sum_{k=1}^{N}\left[\phi_{k}^{\mathrm{kin}}\left(\cdot \right)+\Phi_{k}^{\mathrm{kin}}\left(\begin{array}{c} h\left[k\right]-{\;}^{\mathrm{cen}}h\left[k\right]\\ p_{i}\left[k\right]-{\;}^{\mathrm{cen}}p_{i}\left[k\right] \end{array}\right)\right]$$

(11b) $$h\left[k\right]=A_{G}\left(q\left[k\right]\right)\dot{q}\left[k\right]$$

(11c) $$\dot{q}\left[k+1\right]-\dot{q}\left[k\right]=\ddot{q}\left[k\right]\Delta t,q\left[k+1\right]-q\left[k\right]=\dot{q}\left[k\right]\Delta t$$

(11d) $${\dot{q}}_{\min}\leq \dot{q}\left[k\right]\leq {\dot{q}}_{\max},q_{\min}\leq q\left[k\right]\leq q_{\max}$$

(11e) $${\dot{q}}_{L}\left[k\right]={\dot{q}}_{R}\left[k\right]$$

(11f) $$\left(p_{i}^{xy}\left[k\right]-p_{i}^{xy}\left[k\right]\right)c_{i}\left[k\right]=0$$

(11g) $$p_{i}^{z}\left[k\right]-h_{terrain}\geq 0$$

(11h) $$\ell^{\min}\leq {∥p\left[k\right]-r\left[k\right]∥}_{2}\leq \ell^{\max}$$

The quadratic cost (see Equation (11a)) includes a user-defined cost $\phi_{k}^{\mathrm{kin}}\left(\cdot \right)$ and consensus cost $\Phi_{k}^{\mathrm{kin}}$. In detail, the user-defined cost $\phi_{k}^{\mathrm{kin}}\left(\cdot \right)$ is used to minimize joint acceleration, which can be expressed as follows:

(12) $$\phi_{k}^{\mathrm{kin}}\left(\cdot \right)={∥\ddot{q}\left[k\right]∥}_{Q_{q}}^{2}$$

where $Q_{q}\in R^{n+6\times n+6}$ denotes the weight matrix.

The consensus cost $\Phi_{k}^{\mathrm{kin}}$ penalizes deviations in the CoM position ${\;}^{\mathrm{cen}}r$, centroidal momentum ${\;}^{\mathrm{cen}}h$, and foot positions ${\;}^{\mathrm{cen}}p_{i}$ from the solution of the centroidal momentum, which enforces consistency between the dynamic and whole-body kinematic problems. It can be set as follows:

(13) $$\Phi_{k}^{\mathrm{kin}}\left(\cdot \right)={∥h\left[k\right]-{\;}^{\mathrm{cen}}h\left[k\right]∥}_{Q_{fh}}^{2}+{∥r\left[k\right]-{\;}^{\mathrm{cen}}r\left[k\right]∥}_{Q_{fr}}^{2}+{∥p_{i}\left[k\right]-{\;}^{\mathrm{cen}}p_{i}\left[k\right]∥}_{Q_{fp}}^{2}$$

where h, r, and $p_{i}$ are calculated by the floating base kinematics, and $Q_{fh}\in R^{6\times 6}$, $Q_{fr}\in R^{3\times 3}$, and $Q_{fh}\in R^{3\times 3}$ are weight matrices.

**Integration constraint**: The constraints of the whole-body kinematic include the dynamical constraint (see Equation (11b)) and integration constraint (see Equation (11c)).

**Joint constraint**: The joint position should be limited within $q_{\min}$ and $q_{\max}$ to avoid exceeding the joint position limits. Similarly, joint velocities should also be restricted (Equation (11d)). More importantly, $q_{L}$ represents the joint positions of the left leg and the left arm, and $q_{R}$ represents the joint positions of the right leg and the right arm. The joint velocities of ${\dot{q}}_{L}$ and ${\dot{q}}_{R}$ should be consistent because of the symmetry of the robot (Equation (11e)).

**Terrain constraint**: The tangential displacement between the two knot points must be zero when the foot does not contact the ground. This constraint disappears during the flight phase. Equation (11f,g) guarantee that the foot position remains above the terrain surface.

**Distance constraint**: The distance constraint is expressed in Equation (11h). By tracking the reference variables provided by the centroidal dynamics optimization, a high vertical jumping trajectory can be obtained through whole-body kinematic optimization.

## 3. Simulation and Experimental Results

### 3.1. Validation Setup

The humanoid robot platform, model parameters, and solution for the optimization problem are described below.

The proposed optimization method was demonstrated on a humanoid robot, as shown in Figure 2. This humanoid robot has a total mass of 42.6 kg, a height of 1.5 m, and 14 degrees of freedom. The hip and arm joints are driven by a quasi-drive actuator composed of a brushless DC motor and a harmonic reducer, whereas the knee and ankle joints are driven by a brushless DC motor with ball screw structures. These joints can smoothly and instantaneously switch between position mode and torque mode [17]. There are two six-axis force/torque sensors (M3714B2, Sunrise Instruments, Canton, MI, USA) that measure contact force/torque. Furthermore, a Modbus communication system is established using EtherCAT, with a control cycle time of 1 ms. The model parameters of the humanoid robot are derived from the robot CAD model. Considering the large number of robot connecting rods, the masses and inertias of these links are not listed separately.

The FMINCON function in MATLAB is used to construct and solve the single sequential kino-dynamic trajectory optimization problem for jumping behaviors [36]. This tool allows for flexibility in defining system dynamics, path constraints, and boundary constraints as per user requirements. Additionally, the optimization program is executed on a quadcore computer (Intel Core i7-7700, 3.60 GHz).

### 3.2. Numerical Optimization

Using the single sequential kino-dynamic trajectory optimization, a vertical jumping trajectory with a vertical height of 0.5 m (foot lifting distance) was generated that includes both the launching and flight phases. The discrepancies in consistency between the centroidal dynamics and whole-body kinematics, especially regarding momentum and foot position, are discussed in detail. In addition, the main parameters used in the optimization process can be found in Table 1.

**Figure 3:**
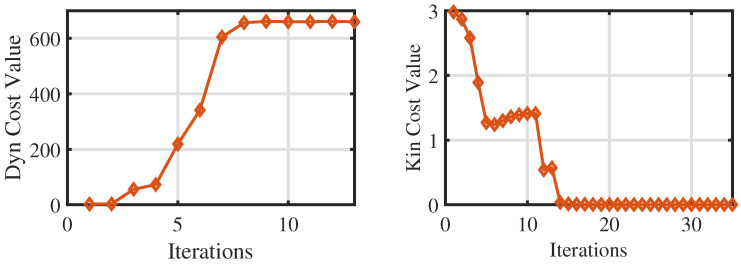
The trend of the cost value of the centroidal dynamics and whole-body kinematics with iterations.

Table 2 lists the main parameters for the numerical optimization, such as the number of variables and the number of equality constraints, and also presents the solving times for both optimization problems. The solving times for centroidal dynamics and whole-body kinematics optimization are 0.230 s and 3.2 s, respectively. In addition, the trend of the cost value with iterations is shown in Figure 3. As the number of iterations increases, the cost value tends to be stable, indicating that the optimization problem is approaching convergence. Therefore, it is evident that this optimization process is highly efficient and takes little time.

**Figure 4:**
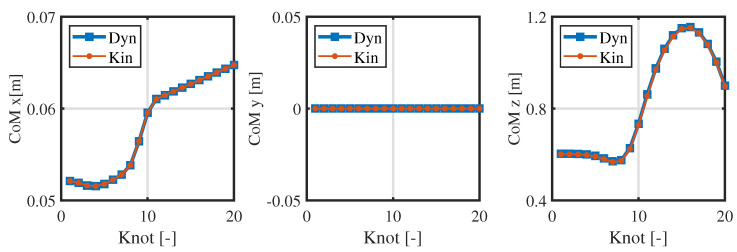
Consistency in the CoM of the centroidal dynamics and whole-body kinematics.

**Figure 5:**
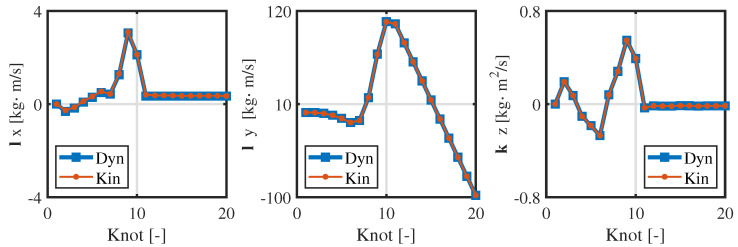
Consistency in the momentum of the centroidal dynamics and whole-body kinematics.

**Figure 6:**
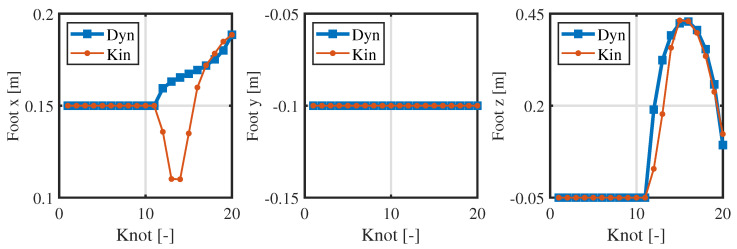
Consistency in the foot position of the centroidal dynamics and whole-body kinematics at the contact point $c_{1}$.

From Figure 4 and Figure 5, the consistency of the momentum and CoM at each knot is high. Ensuring the consistency of momentum and CoM is key when bridging the centroidal dynamic and whole-body kinematic optimization, and hence, the relevant weight matrices $Q_{fr}$ and $Q_{fh}$ in Equation (13) are adjusted to maintain momentum consistency. Notably, the consistency of the foot position is poor (see Figure 6), and even the motion trends in the *x*-direction are quite different. This observed phenomenon can be attributed to the centroidal dynamics model, which focuses primarily on the relative position between the CoM and the foot position without introducing whole-body kinematic constraints. Because the optimized foot position might not meet the whole-body kinematic constraints, it is treated merely as a reference trajectory during the whole-body optimization, and high consistency is not pursued.

**Figure 7:**
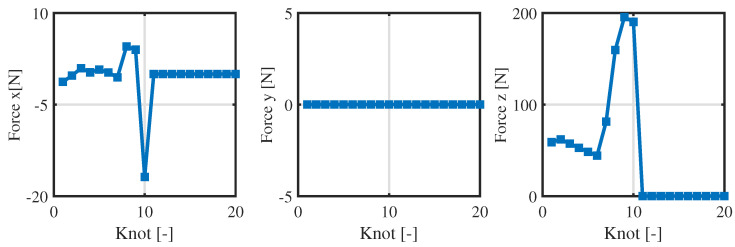
Optimized contact forces of contact point $c_{1}$ in the centroidal dynamics optimization.

Figure 6 and Figure 7 presents the positions and contact forces at the contact point $c_{1}$. When this point is touching the ground, there is obviously no position sliding and the normal contact force exists; during the flight phase, the contact force is zero and the position changes. These fully meet the complementarity conditions (see Equations (2h,i) and (11f)). This result verifies the effectiveness of the complementary conditions and its combination with the re-specified contact sequence. Finally, the whole-body trajectory including the launching and flight phases is obtained.

**Figure 13:**
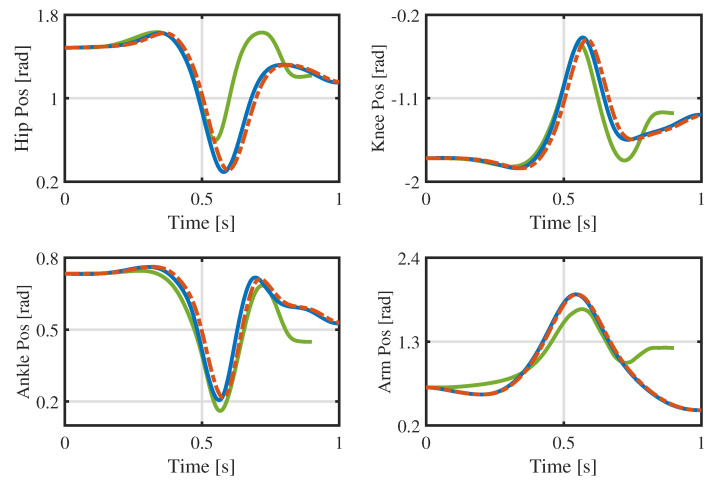
Joint angles in the launching and flight phases of the experiment.

To sum up, the whole-body trajectory of the vertical jump can be optimized using a single sequential optimization, and it is an efficient solution while saving time. After solving the optimization problem, the state values and control values that satisfy the constraints of the centroidal dynamics and whole-body kinematics are obtained. Subsequently, the whole-body trajectory is derived by utilizing a spline function to interpolate the states q between two knot points, which were used for the simulation and experiment (see Figure 13).

### 3.3. Simulations

The effectiveness of the optimized trajectory was verified using CoppeliaSim dynamics software. During the launching and flight phases, the whole-body trajectory was performed in the joint position mode of the robot. Then, the joints smoothly switched from position mode to torque mode. For the landing phase, a landing controller that had been designed for our team’s previous research [17] was adopted. Here, the CoM trajectory was derived from the centroidal dynamics optimization.

**Figure 8:**
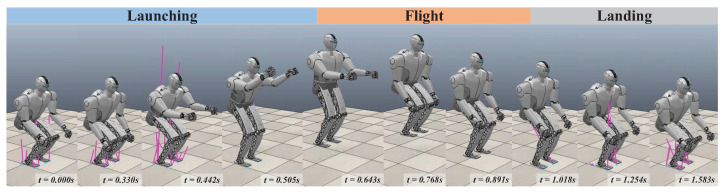
Snapshot of the simulation in the CoppeliaSim simulator with the optimized trajectory (Supplementary Materials).

**Figure 9:**
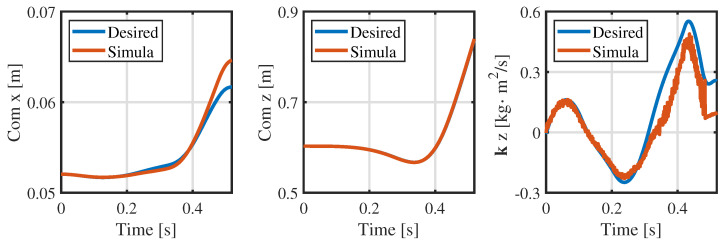
CoM position and angular momentum during the launching phase in the simulation.

Figure 8 shows a snapshot of the jumping simulation. The humanoid robot was able to achieve a stable launch as the planned jumping motion. Figure 9 illustrates the tracking of the CoM position and angular momentum in the launching phase. Obviously, the CoM tracks well in the *z* and *x* directions, and the maximum tracking error is less than 3 mm when taking off in the *x* direction, which is well within tolerance. In addition, the angular momentum around the *y*-axis also has a good tracking effect, and the angular momentum is close to the reference value at the moment of take-off.

**Figure 10:**
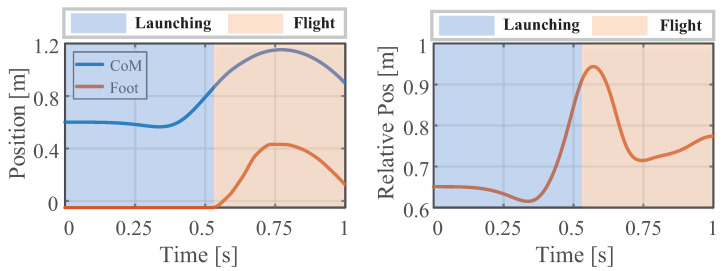
Absolute and relative positions of the humanoid robot’s CoM and foot under vertical jump conditions.

Additionally, the robot has an obvious leg retraction motion in the first half of the flight phase, which results in a high jump (foot lifting distance). Figure 10 depicts the absolute positions of the CoM and foot as well as their relative distance. The CoM rises by 0.30 m, and the foot retracts concurrently by 0.18 m. This coordinated motion enables the robot to achieve a jump height of 0.48 m from the ground. According to Equation (4) in Section 2.2, the relative distance between the foot and CoM can be adjusted by changing the weight matrix $Q_{ce}$. In addition, the arm’s motion can be leveraged more effectively by adjusting the weight matrix $Q_{q}$, which prevents excessive trunk bending and ensures a stable posture during both the launching and flight phases.

### 3.4. Experiments

**Figure 11:**
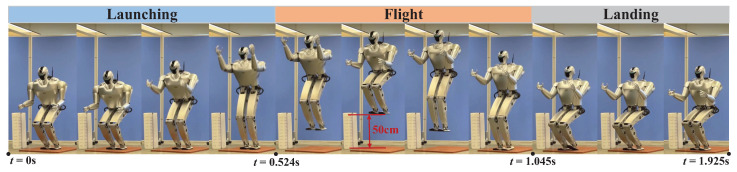
Snapshot of the experiment with a 0.5 m (foot lifting distance) vertical jump (Supplementary Materials).

The same whole-body trajectory used in the simulation was also executed on a real humanoid robot, as depicted in Figure 11. Clearly, the robot can achieve stable launching similar to the simulation, then retract its legs during the flight phase to achieve a 0.5 m (foot lifting distance) vertical jump, and subsequently maintain a stable posture until landing.

**Figure 12:**
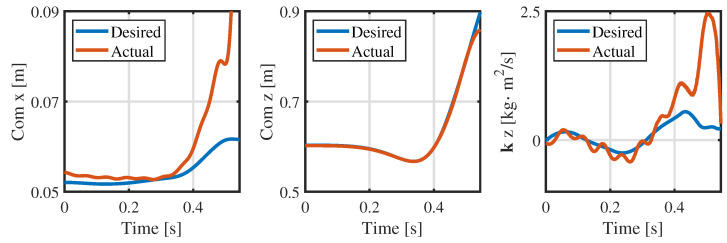
CoM position and angular momentum in the launching phase in the experiment.

Figure 12 illustrates tracking of the CoM position and momentum in the launching phase. The joint angle and velocity are filtered by a low-pass filter, and the CoM position and momentum data are effectively computed through the forward kinematic and momentum equation. The actual CoM position tracks well in the *z* direction. Moreover, the tracking effectiveness in the *x* direction is not satisfactory, but the general trend is roughly the same. More importantly, the actual momentum around the *y*-axis is tracked satisfactorily in the initial 0.4 s, and then the actual angular momentum rises rapidly to about 2.5 kg · m^2^/s and then rises sharply to 0.2 kg · m^2^/s at the moment of take-off.

**Figure 14:**
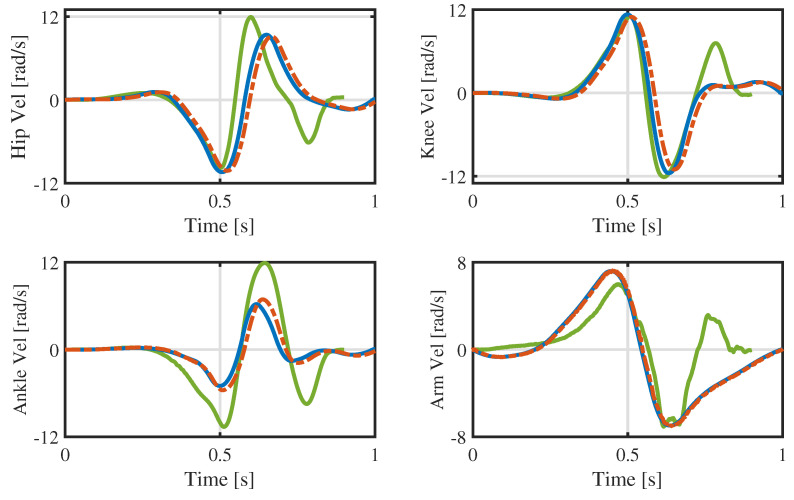
Joint velocities in the launching and flight phases of the experiment.

**Figure 15:**
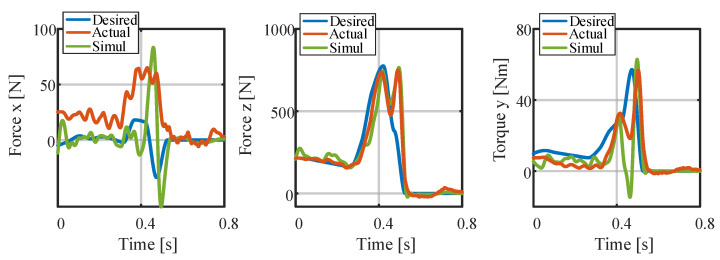
Contact forces in the launching and flight phases of the experiment.

The reason for this phenomenon may be poor tracking of the joint angle and velocity, particularly in the late launching phase. Taking the joint motion of the robot’s left part as an example, Figure 13 and Figure 14 illustrate, respectively, the tracking performance of the joint angle and velocity in the launching and flight phases. There are certain tracking errors in the angles and velocities of the hip, knee, and ankle joints, coupled with the fact that the actual contact force in the *x*-direction is much larger than desired (the desired contact forces at the contact surface vertices are converted into a six-dimensional force at the footplate [33], and the results are shown in Figure 15), which together make the robot move forward in the *x*-direction. As a result, the pressure center of the foot gradually moves toward the toes, causing the heel to lift. The heel is raised about 2∼3 cm in height, as can be seen in Figure 11. This may lead to fluctuation in the contact force and contact torque at about *s*, which, in turn, causes poor tracking of the angular momentum.

Figure 13 and Figure 14 also compare the optimized whole-body trajectory with those of our previous research [17]. (The joint trajectories collected in the dynamic simulation are combined with reaction momentum control (RMC) [37] to generate the whole-body trajectory for the launching and flight phases. All jump heights are 50 cm, and the joint trajectories are represented by the icon labeled ’TRO’.) The maximum speeds of the hips, knees, and ankles decrease by varying degrees, and the velocity curve is smoother. Therefore, the proposed optimization method not only generates more usable trajectories but also obtains them more quickly.

The experimental results show that the optimized trajectory successfully realizes the desired vertical jumping motion during the launching and flight phases, and hence, the effectiveness of the proposed method has been verified.

## 4. Conclusions

High vertical jumping motion directly reflects a humanoid robot’s extreme motion capabilities and enables it to leap over obstacles. This article proposed a novel sequential dynamic trajectory optimization method that sequentially optimizes the centroidal dynamics and whole-body kinematics to solve the whole-body trajectory for high vertical jump motions. The proposed method is an efficient solution strategy that significantly reduces the solving time: giving a total optimization time of approximately 3.43 s. To validate the effectiveness of the single sequential kino-dynamic trajectory optimization, the optimized whole-body trajectory was verified through simulation and experimentation on a humanoid robot platform. The results show that the CoM position and momentum during the launching phase are satisfactorily tracked; the humanoid robot can achieve a stable vertical jump of 0.5 m. After comparing the optimized trajectory with the trajectory collected in the dynamic simulation and RMC, we found that the maximum joint velocity decreases by varying degrees, demonstrating that the optimized trajectory is more suitable for jump motion planning. The proposed method can be applied to a bipedal robot, and it is suitable for humanoid robots, which have high degrees of freedom. In the future, the proposed method will be enhanced to achieve online whole-body trajectory optimization and provide more practical whole-body trajectories combined with environment-aware information. Additionally, we plan to achieve more agile kinds of jump motions, such as a back flip.

## Figures and Tables

**Table 1:** Main parameters for the single sequential kino-dynamic trajectory optimization.

Main Parameter	Value
Knot points, N	20
Motion duration, T	1 s
Contact sequence of $c_{1}$, $c_{2}$, … and $c_{8}$	[1,…,1,0,0,0,0,0,0,0]
Optimality tolerance	<1 × 10^−5^
$Q_{cf}$	diag (0.001, 0.001, 0.001, *…*, 0.001, 0.001, 0.001)
$Q_{cr}$	diag (40, 40, 40)
$Q_{ch}$	diag (30, 10, 10, 10, 50, 10)
$Q_{ce}$	diag (0.01, 0.01, 0.01, …, 0.01, 0.01, 0.01)
$Q_{q}$	diag (0.0001, 0.0001, 0.0001, …, 0.0001)
$Q_{fh}$	diag (1, 1, 1, 1, 1, 1)
$Q_{fr}$	diag (0.01, 0.01, 0.01)
$Q_{fp}$	diag (0.02, 0.02, 0.02, …, 0.02, 0.02, 0.02)
$Q_{fe}$	diag (0.01, 0, 0.001, …, 0.01, 0, 0.001)

**Table 2:** Numerical optimization of main parameters.

Optimization Project	Centroidal Dynamics	Whole-Body Kinematics
Number of variables	720	1200
Duration of motion	1 s	1 s
Number of equality constraints	1036	720
Number of inequality constraints	3102	4250
Solving time	0.230 s	3.2 s
Number of iterations	12	35

## Data Availability

No new data were created or analyzed in this study. Data sharing is not applicable to this article.

## Supplementary Materials

The following supporting information can be downloaded at: https://www.mdpi.com/article/10.3390/biomimetics9050274/s1.
